# Ancestral Function and Diversification of a Horizontally Acquired Oomycete Carboxylic Acid Transporter

**DOI:** 10.1093/molbev/msy082

**Published:** 2018-04-25

**Authors:** Fiona R Savory, David S Milner, Daniel C Miles, Thomas A Richards

**Affiliations:** Living Systems Institute, School of Biosciences, College of Life and Environmental Sciences, University of Exeter, Exeter, United Kingdom

**Keywords:** osmotrophy, gene transfer, transporter, neofunctionalization, ancestral sequence reconstruction

## Abstract

Horizontal gene transfer (HGT) can equip organisms with novel genes, expanding the repertoire of genetic material available for evolutionary innovation and allowing recipient lineages to colonize new environments. However, few studies have characterized the functions of HGT genes experimentally or examined postacquisition functional divergence. Here, we report the use of ancestral sequence reconstruction and heterologous expression in *Saccharomyces cerevisiae* to examine the evolutionary history of an oomycete transporter gene family that was horizontally acquired from fungi. We demonstrate that the inferred ancestral oomycete HGT transporter proteins and their extant descendants transport dicarboxylic acids which are intermediates of the tricarboxylic acid cycle. The substrate specificity profile of the most ancestral protein has largely been retained throughout the radiation of oomycetes, including in both plant and animal pathogens and in a free-living saprotroph, indicating that the ancestral HGT transporter function has been maintained by selection across a range of different lifestyles. No evidence of neofunctionalization in terms of substrate specificity was detected for different HGT transporter paralogues which have different patterns of temporal expression. However, a striking expansion of substrate range was observed for one plant pathogenic oomycete, with a HGT derived paralogue from *Pythium aphanidermatum* encoding a protein that enables tricarboxylic acid uptake in addition to dicarboxylic acid uptake. This demonstrates that HGT acquisitions can provide functional additions to the recipient proteome as well as the foundation material for the evolution of expanded protein functions.

## Introduction

Horizontal gene transfer (HGT) involves the transfer of genetic material between reproductively isolated lineages and can allow recipient organisms to adapt to a novel lifestyle or to exploit a new ecological niche (Doolittle 1999; Jain et al. 2003; Keeling and Palmer 2008; Richards and Talbot 2013). HGT is highly prevalent in prokaryotes, and is becoming increasingly recognized as an important mechanism driving evolutionary innovation and adaptation in eukaryotes (Keeling and Palmer 2008). For instance, horizontal acquisitions of putative virulence genes have been reported in fungi (Friesen et al. 2006; Marcet-Houben and Gabaldon 2010; Slot and Rokas 2011; Gardiner et al. 2012; Wisecaver et al. 2014; Zhao et al. 2014), and HGT genes involved in metabolism in anaerobic environments have been detected in the genomes of anaerobic protists and fungi (Gojkovic et al. 2004; Ricard et al. 2006; Slamovits and Keeling 2006; Hall and Dietrich 2007; Eme et al. 2017). However, putative functions of horizontally acquired genes in eukaryotes have typically been inferred based on shared sequence identity to characterized genes of distantly related model organisms. Few studies have determined the functions of HGT genes experimentally (Friesen et al. 2006; Gardiner et al. 2012; Kirsch et al. 2014; Zhao et al. 2014; Alexander et al. 2016), or investigated postacquisition functional divergence from an ancestral state (Aoki 2004) approximating the HGT acquired gene. This constrains our ability to understand how HGT events can play a role in determining the ecology and cellular functions of the recipient taxa and limits our understanding of how horizontally acquired genes may have contributed to the evolution of recipient lineages.

Among the supported cases of HGT in eukaryotes are a variety of genes encoding putative transporter proteins, which mediate the translocation of molecules across cell membranes (Richards et al. 2006, 2009, 2011; Slot and Hibbett 2007; Galeote et al. 2010; Marcet-Houben and Gabaldon 2010; McDonald et al. 2012; Coelho et al. 2013; Schönknecht et al. 2013; Marsit et al. 2016; Major et al. 2017). Gaining novel transporter genes via HGT may be of particular importance to osmotrophic organisms, which feed by secreting depolymerising enzymes into the external environment to break down complex molecules, and then importing the resulting subunits into the cell through specialized membrane transporter proteins (Richards and Talbot 2013). Specifically, horizontal acquisition of transporter genes could allow osmotrophs to colonize new niches by facilitating the use of nutrients that were previously inaccessible (e.g., Slot and Hibbett 2007; Galeote et al. 2010; Coelho et al. 2013), and/or outcompete other organisms that are present in the same environment (Richards and Talbot 2013). However, the substrate specificities of many transporters are poorly annotated in genomic databases and members of the same protein family can often transport a variety of different substrates (Moran et al. 2016), making it difficult to identify the subsequent ecological role of horizontally acquired transporter proteins in recipient lineages.

Oomycetes are eukaryotic microbes that feed by osmotrophy. They include a diversity of forms, from free-living saprotrophs, which obtain nutrients from decaying matter, to obligate and opportunistic pathogens of plants and animals. Oomycetes superficially resemble fungi, but belong to the Stramenopile (Heterokonta) phylum, and descended from a phagotrophic and possibly photosynthetic ancestor (Cavalier-Smith and Chao 2006; but see Stiller et al. 2009, 2014). This radical change of lifestyle and feeding strategy, from an ancestral form that engulfs and digests microbes inside the cell and/or fixes carbon by photosynthesis, to one which breaks down complex molecules in the external environment and imports nutrients into the cell, may have been facilitated by HGT (Torto et al. 2002; Richards et al. 2006, 2011; Richards and Talbot 2013; Misner et al. 2014). Indeed, phylogenetic analyses suggest that a variety of oomycete genes which putatively encode osmotrophy associated proteins, such as secreted depolymerising enzymes and membrane transporters, were horizontally acquired from fungal donors (Richards et al. 2006, 2011; Savory et al. 2015). The majority of these HGTs are specific to plant pathogenic oomycetes given current genome sampling (Richards et al. 2011; Soanes and Richards 2014; Savory et al. 2015) and may provide the means to invade and obtain nutrients from the host. However, one HGT transporter gene family appears to have been acquired early in the oomycete radiation, prior to the divergence of the major oomycete lineages, as orthologues have been detected in the genomes of Peronosporaleans, which are predominantly plant pathogenic, and Saprolegnialeans, which include opportunistic pathogens of invertebrates, fish and amphibians, as well as nonpathogenic saprobes (Richards et al. 2006, 2011; Savory et al. 2015). The ancient acquisition of this HGT gene family and the retention of orthologues in distinct oomycete lineages is indicative of an important adaptive role, which may have been associated with the transition to an osmotrophic lifestyle (Richards et al. 2006; Soanes and Richards, 2014). Based on PFAM and CDD (Marchler-Bauer et al. 2005) analyses, the HGT gene family was assigned to the Major Facilitator Superfamily (MFS), and the proteins were putatively annotated as monosaccharide sugar transporters (Richards et al. 2006, 2011). Here we functionally characterize members of the HGT transporter family and use ancestral sequence reconstruction to confirm that the ancestral protein functions as a transporter, identify substrate ranges across the oomycete gene family, and investigate postacquisition functional divergence during the oomycete radiation.

## Results and Discussion

### HGT and Postacquisition Evolutionary Dynamics

**Fig. 1.**
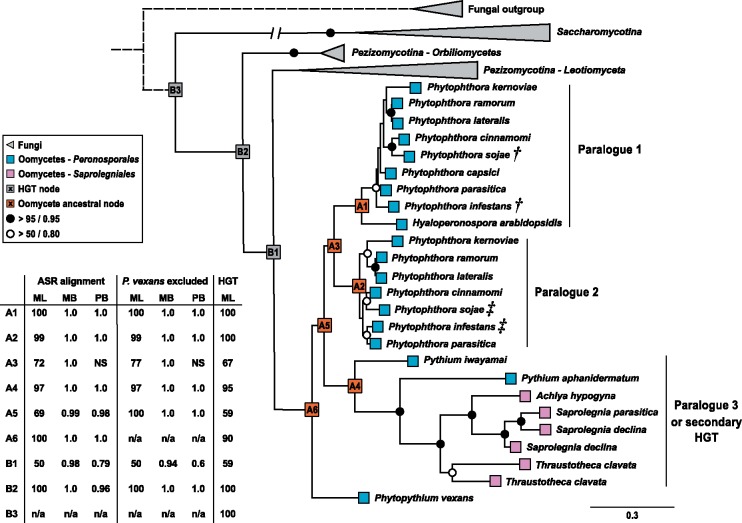
Maximum likelihood phylogeny of oomycete and fungal transporter proteins. The displayed tree is a composite of the reduced taxa tree used for calculating ancestral proteins with a schematic representation of a phylogeny encompassing wider taxonomic groups (i.e., additional outgroup sampling). RaxML bootstrap support values and MrBayes (MB)/Phylobayes (PB) posterior probabilities are tabulated for key internal nodes, including oomycete ancestral nodes A1–A6 and “HGT” nodes B1–B3, which show oomycete sequences grouping within the ascomycete fungal clade (NS—node not supported). Support values are tabulated for phylogenies inferred from 1) the alignment used for ancestral sequence reconstruction (ASR), 2) the ASR alignment following removal of the *P. vexans* sequence (see text for details), and 3) an extended alignment containing additional *Saccharomycotina* sequences and more distant fungal outgroup sequences (HGT) (see supplementary fig. S1, Supplementary Material online). In the latter case, only RaxML bootstrap support values are reported. Support values for other nodes are marked if bootstrap values and posterior probabilities are above 95% and 0.95, respectively (shaded circles), or 50% and 0.8, respectively (open circles). Symbols correspond to oomycete transporter proteins that are upregulated prior to (†) and during (‡) infection (Torto-Alalibo et al. 2007; Roy et al. 2013). Fungal nodes were collapsed and the branch connecting the *Saccharomycotina* outgroup was truncated for tree display (full ML and Bayesian phylogenetic trees are presented in supplementary figs. S1–S4, Supplementary Material online). The additional fungal outgroup, represented by a dashed line, was inferred from supplementary figure S1, Supplementary Material online and previous studies (Richards et al. 2006, 2011).

We used maximum likelihood (ML) and Bayesian methods to reconstruct the phylogeny of the oomycete HGT transporters from an alignment of oomycete and fungal protein sequences (supplementary data sets S1 and S2, Supplementary Material online). The alignment contained sequences from oomycetes with different lifestyle strategies, including obligate biotrophs, hemibiotrophs, necrotrophs, and nonpathogenic saprotrophs, and a selection of orthologous fungal sequences that were identified in similarity searches. The fungal sequences corresponded to the Pezizomycotina subphylum, previously inferred as the HGT donor lineage (Richards et al. 2006, 2011), as well as the Saccharomycotina subphylum, and a set of more distant fungal paralogues was included as an outgroup. Orthologues were absent in nonoomycete Stramenopiles, including *Hyphochytrium catenoides*, a free-living sister of the oomycetes (Leonard et al. 2018). No major topological differences were detected among trees generated using different phylogenetic approaches. The analyses yielded a phylogeny with strong statistical support for the placement of oomycete sequences within the fungal lineage (fig. 1 and supplementary figs. S1–S4, Supplementary Material online), providing further confirmation that the ancestral oomycete HGT transporter gene was horizontally acquired from a fungal donor. The placement of oomycete sequences within the Pezizomycotina lineage had moderate statistical support (fig. 1 and supplementary figs. S1–S4, Supplementary Material online), consistent with the hypothesis that the fungal donor belonged to the Pezizomycotina subphylum or was a close relative of the Pezizomycotina (Richards et al. 2006, 2011).

The HGT transporter sequences were detected in the majority of oomycetes for which genome data were available (fig. 1 and supplementary figs. S1–S4, Supplementary Material online) (Savory et al. 2015), suggesting that the transporter proteins have an important function in these microbes which is conserved across lineages with different lifestyle strategies. However, our phylogenetic analyses revealed unexpected relationships that are not consistent with the oomycete species phylogeny (e.g., McCarthy and Fitzpatrick 2017; Ascunce et al. 2017), as sequences corresponding to two Peronosporalean *Pythium* species repeatedly grouped with Saprolegniales sequences with strong statistical support (fig. 1 and supplementary figs. S1–S4, Supplementary Material online). Additionally, the *Phytopythium vexans* (previously *Pythium vexans*) HGT transporter sequence appears to have diverged prior to the other oomycete sequences, yet this species occupies a phylogenetic position which is immediately basal to the *Phytophthora* and *Hyaloperonospora arabidopsidis* clade in species phylogenies. These results could reflect artifacts of the sequences and/or phylogenetic reconstructions within the oomycete radiation. Alternatively, they may be indicative of differential patterns of loss following postacquisition gene expansion and/or a secondary HGT event, whereby a Saprolegnialean ancestor acquired a transporter sequence from a Peronosporalean donor. In the latter case, this would imply a more recent acquisition of the HGT transporter gene family from fungi, after the split between the Peronosporalean and Saprolegnialean lineages, which is estimated to have occurred around 200 Ma (Matari and Blair 2014). HGT transporter sequences were not detected in the genomes of Albuginales or Aphanomyces species (Savory et al. 2015, fig. 1*A*: HGT gene family 3), which diverged early in the Peronosporalean and Saprolegnialean radiations, respectively (Petersen and Rosendahl 2000; McCarthy and Fitzpatrick 2017). This could potentially support the occurrence of a secondary HGT event. However, further genome sequencing of Pythiales, Saprolegniales and early branching oomycetes would provide greater insight into the taxonomic distribution of the gene family, and allow us to make more robust inferences regarding the phylogenetic positions and postacquisition evolutionary dynamics of the HGT transporter sequences.

The genomes of plant pathogenic, hemibiotrophic *Phytophthora* species typically contain two HGT transporter paralogues (fig. 1 and supplementary figs. S1–S4, Supplementary Material online), indicating that a duplication event occurred prior to the radiation of this genus (only a partial sequence was detected for one *P. capsici* paralogue, perhaps reflecting loss of one gene copy or incomplete genomic sequence data). The retention of two intact gene copies in multiple *Phytophthora* species suggests that both transporter proteins are functional. Transcriptome data from two *Phytophthora* species reveal that the paralogues have developmental stage-specific patterns of expression; whilst one paralogue is upregulated in zoospores and/or cysts, the other is upregulated in hyphae (Torto-Alalibo et al. 2007; Roy et al. 2013) (fig. 1). Two paralogues were also detected in the genomes of *Thraustotheca clavata*, a free-living saprotroph, and *Saprolegnia declina*, an opportunistic pathogen of aquatic animals (fig. 1 and supplementary figs. S1–S4, Supplementary Material online). However, these paralogues appear to have arisen from recent lineage-specific gene duplication events.

### Homology with “Jen” Carboxylic Acid Transporters

Fungal orthologues of the oomycete HGT transporters belong to the Sialate: H^+^ Symporter (SHS) family and are referred to as “Jen” proteins (Casal et al. 1999, 2008). Jen proteins share homology with an *Escherichia coli* monosaccharide transporter (Saier 2000) but have been shown to preferentially transport carboxylic acids in Saccharomycotina yeasts (Casal et al. 1999; Soares-Silva et al. 2004, 2007, 2011, 2015; Vieira et al. 2010; Dulermo et al. 2015; Guo et al. 2015) and the Pezizomycotina fungus *Aspergillus nidulans* (Sá-Pessoa et al. 2015). Many fungal genomes contain multiple Jen paralogues, and, in some cases, these have nonoverlapping substrate specificities. For instance, in some Saccharomycotina yeasts, Jen1 proteins transport monocarboxylic acids, such as lactic acid and pyruvic acid, whilst Jen2 proteins transport dicarboxylic acids, such as succinic acid and malic acid (Casal et al. 1999; Soares-Silva et al. 2004, 2007, 2011, 2015; Lodi et al. 2004, 2007; Queirós et al. 2007; Vieira et al. 2010). Conserved residues which are critical determinants of substrate specificity have been identified within transmembrane domains that form the substrate translocation pathway of the *Saccharomyces cerevisiae* Jen1 transporter (Soares-Silva et al. 2011). Residues that are required for dicarboxylic acid uptake are present in Pezizomycotina Jen proteins (Lodi et al. 2007; Sá-Pessoa et al. 2015) and the oomycete HGT transporters (supplementary fig. S5, Supplementary Material online). However, the two *A. nidulans* Jen orthologues (the only Pezizomycotina Jen proteins that has been functionally characterized) both have the capacity to transport mono- and dicarboxylic acids, albeit with greater affinity for dicarboxylic acids (Sá-Pessoa et al. 2015). This raises the possibility that the ancestral oomycete HGT transporter gene encoded a promiscuous protein, capable of transporting a range of mono- and dicarboxylic acid substrates.

### The Oomycete HGT Transporters Are Functional Carboxylic Acid Transporters

To investigate the function of the ancestral oomycete HGT transporter and postacquisition functional divergence, we used an empirical Bayes approach (Yang et al. 1995) to reconstruct ML protein sequences at key ancestral nodes using a reduced alignment (supplementary data set S3, Supplementary Material online) and corresponding ML phylogeny (supplementary fig. S2, Supplementary Material online). This approach calculates the likelihood of all possible ancestral residues at each site in the protein sequence, and yields a single sequence, for each node of interest, which contains ML states at all sites. The ML ancestral sequence represents the best estimate of the true ancestral sequence given the alignment, the phylogeny and the model of sequence evolution. Ancestral sequence reconstruction algorithms are reported to infer ancestral sequences with high accuracy (Williams et al. 2006; Hanson-Smith et al. 2010; Matsumoto et al. 2015; Randall et al. 2016) and can be reasonably robust to uncertainty associated with the underlying phylogeny and the evolutionary model (Thomson et al. 2005; Chang et al. 2007; Hanson-Smith et al. 2010).

Genes encoding a selection of eight extant transporters and five ancestral transporters were codon optimized, synthesized, and expressed in *S. cerevisiae* for phenotypic characterization. The extant transporters corresponded to oomycetes representing a variety of lifestyle strategies, including obligate biotrophs (*H. arabidopsidis*), hemibiotrophs (*Phytophthora infestans* and *Phytophthora parasitica*), saprotrophs and opportunistic necrotrophs (*Pythium aphanidermatum* and *S. declina*), and free-living saprotrophs (*T. clavata*). The ancestral transporters included the optimal ML protein sequence reconstruction (here named the primary ancestral protein) of each *Phytophthora* paralogue (A1 and A2), the ancestor of the *Phytophthora* paralogues prior to duplication (A3), the ancestor of the Saprolegniales and *Pythium* clade (A4), and an ancient oomycete ancestral form (A5). For a particular site in an ancestral protein sequence, the posterior probability of an amino acid state is derived from the likelihood that the residues observed in all extant sequences included in the alignment would have evolved given that state, the phylogeny, and the evolutionary model. Assuming that posterior probabilities accurately reflect the probability that an inferred ancestral state is correct, mean posterior probabilities can be used as a measure of confidence in ancestral sequence reconstructions (Thornton 2004; Hanson-Smith et al. 2010). Overall confidence in the ancestral sequences was high, with mean posterior probabilities ranging from 0.91 (A4) to 0.98 (A2). Although the node prior to the split between *P. vexans* and the other oomycetes (A6 in Figure 1) represents the earliest ancestral oomycete HGT transporter sequence in the phylogenetic reconstruction, we chose not to characterise this protein due to the unexpected phylogenetic position of *P. vexans*. The *P. vexans* sequence was, however, included in the alignment and tree used for ancestral reconstruction, as removal of the sequence had little effect upon statistical support for key nodes in the tree (Figure 1), although resulted in lower overall confidence in the A5 ancestral sequence identified (mean posterior probability 0.88 compared to 0.92 when the *P. vexans* sequence was retained).

Reconstructed ancestral sequences typically contain ambiguously inferred sites, in which two or more ancestral states are statistically plausible. This is particularly the case when ancient divergences are investigated. In this study, the number of residues with plausible alternative states (considered as non-ML states with posterior probabilities >0.2) in transmembrane domains that form the putative substrate translocation pathway was low, ranging from 0 (A2) to 5 (A5) (supplementary fig. S5, Supplementary Material online). As these transmembrane domains contain conserved residues and sequence motifs that influence substrate specificity in fungal orthologues (Soares-Silva et al. 2011), they represent the most likely regions of the sequences in which ambiguous sites could impact ancestral protein function in the oomycete HGT transporters.

**Fig. 2.**
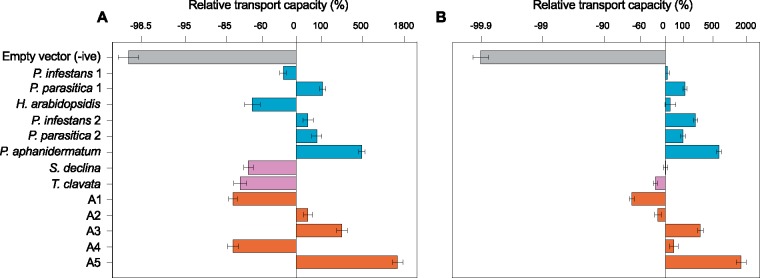
Transport capacity (*V*_max_—the maximum uptake rate) of HGT oomycete transporters. (*A*) ^14^C labeled succinic acid and (*B*) ^14^C labeled malic acid by W303-1A *jen1*Δ *ady2*Δ *S. cerevisiae* strains transformed with an empty vector (negative control) or expressing extant or ancestral oomycete HGT transporter proteins relative to a W303-1A *jen1*Δ *ady2*Δ strain expressing the *C. albicans* JEN2 transporter protein (positive control) (note that the relative transport capacity axis is log scaled). Mean values from three replicated experiments are presented and bars correspond to standard errors.

To determine if extant and ancestral oomycete HGT transporters import carboxylic acids, they were constitutively expressed in the *S. cerevisiae* strain W303-1A *jen1*Δ *ady2*Δ, which lacks native carboxylic acid transporters (Soares-Silva et al. 2007). As the oomycete sequences contain conserved residues that enable the *S. cerevisiae* Jen1 protein to transport dicarboxylic acids (Soares-Silva et al. 2011; supplementary fig. S5, Supplementary Material online), we tested if expression of the oomycete HGT transporters conferred the ability to import radiolabeled succinic acid and malic acid. We detected uptake of ^14^C labeled succinic acid (fig. 2*a* andsupplementary fig. S6, Supplementary Material online) and ^14^C labeled malic acid (fig. 2*b* andsupplementary fig. S7, Supplementary Material online) in the eight W303-1A *jen1*Δ *ady2*Δ strains expressing extant oomycete HGT transporter proteins and the five W303-1A *jen1*Δ *ady2*Δ strains expressing ancestral oomycete HGT transporter proteins. Uptake was also detected in a positive control W303-1A *jen1*Δ *ady2*Δ strain expressing the *Candida albicans* JEN2 protein, a dicarboxylic acid transporter (Vieira et al. 2010), but not in a negative control W303-1A *jen1*Δ *ady2*Δ strain transformed with an empty vector (figs. 2*a* and 2*b* and supplementary figs. S6 and S7, Supplementary Material online). This demonstrates that the oomycete HGT transporter proteins are functional carboxylic acid transporters.

**Fig. 3.**
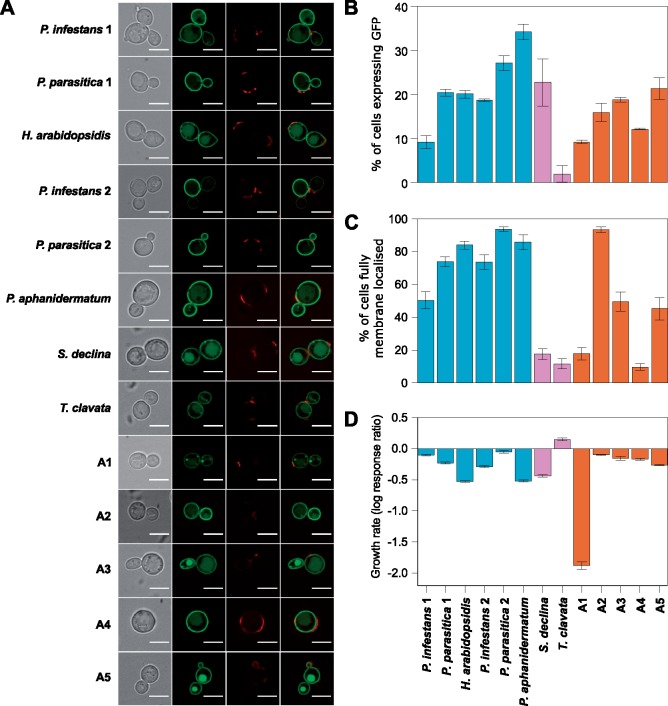
Localization and effects of transporter expression on yeast growth. (*A*) Cellular localization of extant and ancestral (A1–A5) oomycete HGT transporter proteins. Panels display images from the same *S. cerevisiae* W303-1A *jen1*Δ *ady2*Δ cell(s) expressing GFP-transporter fusion proteins; from left to right: bright field, GFP, wheat germ agglutinin (WGA) counterstain of the cell wall, GFP and WGA. (*B*) Percentage of live cells expressing GFP-transporter fusion proteins. (*C*) Percentage of cells in which GFP-transporter fusion proteins were localized only to the plasma membrane rather than to the plasma membrane and additional components of the cell. (*D*) Growth rates of *S. cerevisiae* W303-1A *jen1*Δ *ady2*Δ strains expressing extant or ancestral (A1–A5) oomycete HGT transporter proteins. Rates were estimated from OD_600_ measurements using a logistic population growth equation, and are displayed as log response ratios (log proportional changes in mean growth rates relative to an empty vector W303-1A *jen1*Δ *ady2*Δ strain). In *B*, *C*, and *D*, mean values from three replicated experiments are presented and bars correspond to standard errors.

The capacity to transport ^14^C labeled succinic acid and ^14^C labeled malic acid was similar to or greater than observed for the positive control strain in several strains expressing oomycete HGT transporter proteins (figs. 2*a* and 2*b* and supplementary figs. S6 and S7, Supplementary Material online). However, transport capacity (*V*_max_—the maximum uptake rate) was relatively low in a few strains, particularly for ^14^C labeled succinic acid. For instance, we observed reductions in the capacity to transport ^14^C labeled succinic acid of 29–82% relative to the positive control strain in six strains expressing oomycete HGT transporter proteins (fig. 2*A*). The variation in transport capacity could have arisen due to differences in the efficiency of heterologous expression in *S. cerevisiae* cells. GFP tagging revealed that all of the oomycete HGT transporters localized to the plasma membrane (fig. 3*A*). However, considerable differences in the proportion of cells expressing GFP-transporter fusion proteins were observed (fig. 3*B*), and cellular distributions were highly patchy in some strains, with fusion proteins being localized to additional components other than the plasma membrane in a high proportion of cells (fig. 3*C*). These results are likely to be linked to the observed variation in transport capacity, as strains with the lowest proportions of cells expressing GFP-transporter fusion proteins and/or the lowest proportions of cells in which the fusion proteins were fully localized to the plasma membrane exhibited relatively low capacities to transport ^14^C labeled succinic acid (fig. 2*A*) and/or ^14^C labeled malic acid (fig. 2*B*). With the exception of the strain expressing the *T. clavata* HGT transporter protein, growth rates on glucose were reduced in all strains relative to the negative control (fig. 3*C*). The reduction was most striking for a strain expressing ancestral protein A1, which displayed low transport capacities for both radiolabeled substrates (fig. 2*A* and *B*). Overall these results suggest that a considerable portion of the variation in the capacity to transport ^14^C labeled succinic acid and/or ^14^C labeled malic acid can be explained by differences in the efficiency of heterologous expression and/or toxic effects of heterologous expression in *S. cerevisiae* cells. This limitation of using *S. cerevisiae* as a chassis for expression constrains our ability to detect possible differences in the kinetic properties of the transporter proteins. As such, we predominantly focus on investigating substrate ranges using competitive inhibition assays in which interstrain comparisons are not required.

### Functional Diversification of Substrate Ranges in the Oomycete HGT Transporters

**Fig. 4.**
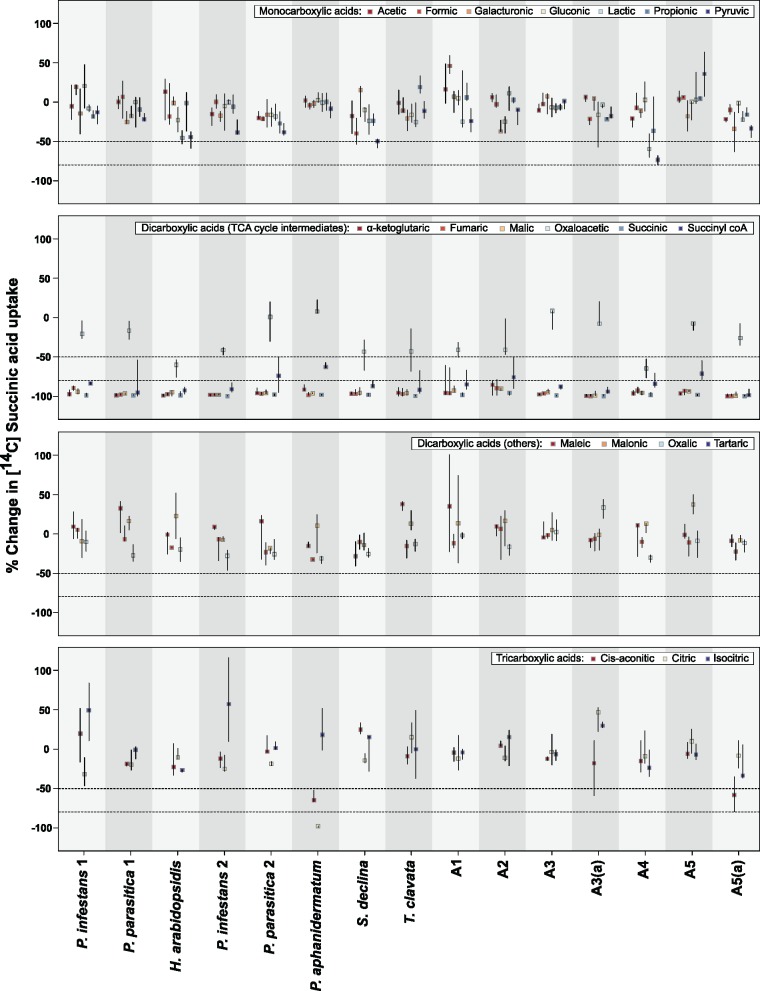
Inhibition of ^14^C labeled succinic acid (50 µM) uptake by addition of nonlabeled substrates (50 mM) in W303-1A *jen1*Δ *ady2*Δ strains expressing extant, ancestral (A1–A5) and alternative ancestral (A3a and A5a) oomycete HGT transporter proteins. Boxes and lines represent medians and interquartile ranges, respectively. Dashed lines correspond to thresholds for considering substrates as moderate or strong inhibitors (upper quartile reduced by 50% or 80%, respectively). Glucose (50 mM) was used as a control (data not displayed).

To further investigate substrate repertoires, we examined the ability of non-labeled carboxylic acids to inhibit ^14^C labeled succinic acid uptake in W303-1A *jen1*Δ *ady2*Δ strains expressing oomycete HGT transporter proteins. Whilst the ability to inhibit succinic acid uptake does not provide definitive confirmation that a substrate can be transported across the plasma membrane, inhibition assays can reveal substrates that transporters bind and potentially transport, allowing us to make inferences about putative substrate ranges. Nonlabeled carboxylic acids were provided at a 1,000-fold greater concentration (50 mM) than ^14^C labeled succinic acid (50 µM) (Soares-Silva et al. 2007) to minimize the probability of discarding substrates with low binding and/or transport capacities, and included seven monocarboxylic acids, ten dicarboxylic acids (including succinyl coA—a combination of succinic acid and coenzyme A) and three tricarboxylic acids (see fig. 4 for substrates used). Glucose (50 mM) was used as a control. We considered a substrate to be a moderate inhibitor or a strong inhibitor if it caused a reduction in ^14^C labeled succinic acid uptake of at least 50% or 80%, respectively, relative to ^14^C labeled succinic acid uptake when no additional nonlabeled substrate was present.

**Fig. 5.**
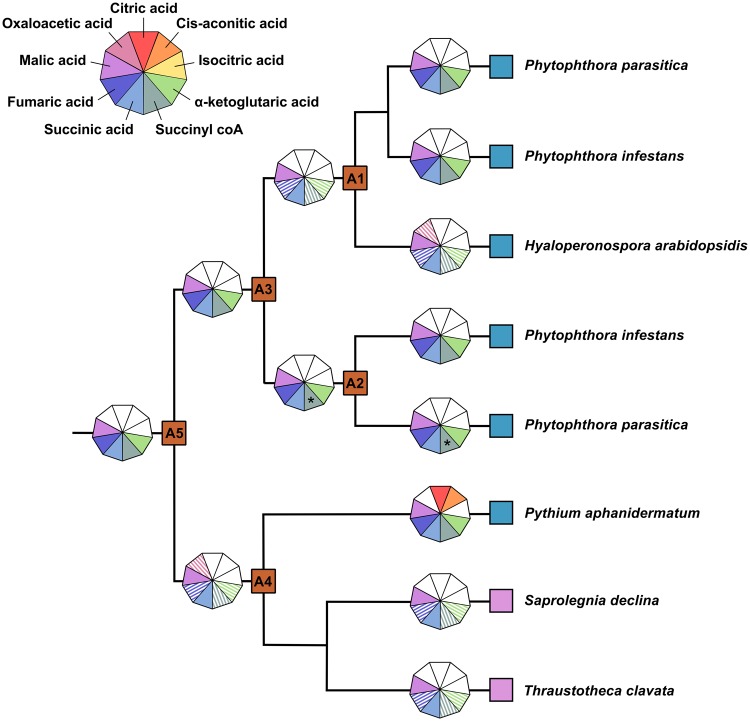
Schematic phylogenetic tree showing retention of oomycete HGT transporter substrate specificity profiles from an ancestral state (A5) through key internal nodes (A1–A4) to extant oomycetes, and expansion of substrate range in *Pythium aphanidermatum*. Substrate specificity profiles are represented by nonagons in which different segments correspond to tricarboxylic acid cycle intermediates and confirmed (radiolabeled uptake assays, see figs. 2 and 6) or putative (competitive inhibition assays, see fig. 4) substrates are coloured. Substrate specificity profiles for strains exhibiting low transport capacity of ^14^C labeled succinic acid are shaded, reflecting the lower confidence that we place in these results (see text for details). Asterisks represent substrates that meet our criteria for consideration as moderate inhibitors by a narrow margin (upper quartile of the response approximately −50%, see fig. 4).

Five nonlabeled dicarboxylic acids inhibited uptake of ^14^C labeled succinic acid in all W303-1A *jen1*Δ *ady2*Δ strains expressing oomycete HGT transporters (figs. 4 and 5), including the six strains with relatively low ^14^C labeled succinic acid transport capacities (fig. 2*A*). The five dicarboxylic acids are all intermediates of the tricarboxylic acid (TCA) cycle, or citric acid cycle, and included succinic acid, malic acid, α-ketoglutaric acid, and fumaric acid, which were strong inhibitors of ^14^C labeled succinic acid uptake in the majority of strains, and succinyl coA, which was a moderate inhibitor in some strains and a strong inhibitor in others (fig. 4). Inhibition of ^14^C labeled succinic acid uptake by additional nonlabeled carboxylic acids was observed only in specific strains. For instance, oxaloacetic acid, which is also a TCA cycle intermediate, was a moderate inhibitor of ^14^C labeled succinic acid uptake in W303-1A *jen1*Δ *ady2*Δ strains expressing the extant *H. arabidopsidis* HGT transporter protein and the A4 ancestral protein (figs. 4 and 5). Pyruvic acid, a monocarboxylic acid, was also a moderate inhibitor of ^14^C labeled succinic acid uptake in the latter strain (fig. 4). However, ^14^C labeled succinic acid transport capacity was relatively low in these strains (fig. 2*A*), meaning that small absolute changes in uptake will be reflected as large relative changes when expressed as proportions or percentages, perhaps leading to incorrect conclusions about potential inhibitory substrates. As such, we do not place strong confidence in these results.

**Fig. 6.**
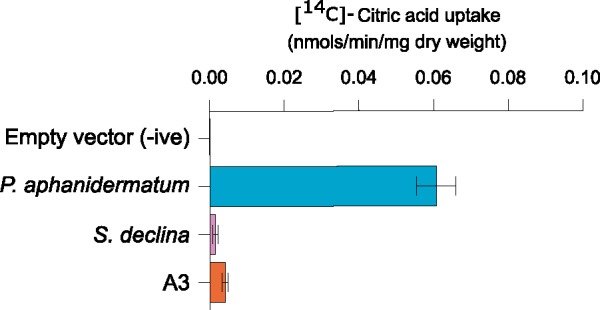
Uptake of 1 mM ^14^C labeled citric acid by W303-1A *jen1*Δ *ady2*Δ *S. cerevisiae* strains transformed with an empty vector (negative control) or expressing the *Pythium aphanidermatum* HGT transporter protein or other representative extant (*S. declina*) and ancestral (A3) oomycete HGT transporter proteins.

A more striking expansion of putative substrate range was detected for the HGT transporter of *Pythium aphanidermatum*, an opportunistic plant pathogen with a broad host range, as citric acid was a strong inhibitor and cis-aconitic acid was a moderate inhibitor of ^14 ^C labeled succinic acid uptake in a W303-1A *jen1*Δ *ady2*Δ strain expressing this protein (figs. 4 and 5). We also detected uptake of ^14 ^C labeled citric acid in this strain (fig. 6), confirming that the *P. aphanidermatum* HGT transporter protein can transport tri-carboxylic acids across the plasma membrane. This gain of function, which could conceivably be linked to the ability of *P. aphanidermatum* to infect a broad range of hosts, may be attributed to changes within transmembrane domains that form the putative substrate translocation pathway, as several residues within these domains were unique to the *P. aphanidermatum* sequence (supplementary fig. S5, Supplementary Material online). These include four sites in transmembrane domain V, in close vicinity to sites which influence substrate specificity in the *S. cerevisiae* Jen1 transporter (Soares-Silva et al. 2011) (supplementary fig. S5, Supplementary Material online). Notably, the residues at two of these sites are hydrophobic in all other extant oomycete HGT transporter sequences and the inferred ancestral sequences, but polar in the *P. aphanidermatum* sequence (supplementary fig. S5, Supplementary Material online), potentially altering the topology of the transporter pore.

We detected no divergence in the range of substrates that the extant and ancestral *Phytophthora* paralogues can transport and/or bind (figs. 4 and 5), and thus no evidence of neofunctionalization [the emergence of novel functions] or subfunctionalization [the division of ancestral functions] (Innan and Kondrashov 2010) in terms of substrate specificity following duplication of the ancestral transporter (A3 in fig. 1). However, we cannot rule out possible differences in transport capacities and/or affinities for particular substrates, as observed for Jen dicarboxylic acid transporter paralogues in *Yarrowia lipolytica* (Dulermo et al. 2015) and *Debaryomyces hansenii* (Soares-Silva et al. 2015). As one *Phytophthora* paralogue is upregulated in zoospores and/or during the cyst stage, whilst the other is upregulated in hyphae (Torto-Alalibo et al. 2007; Roy et al. 2013), such differences could reflect variation in the metabolic requirements of these stages and underlie the retention of two functional paralogues in the *Phytophthora* genus. Though ^14 ^C labeled malic acid Km values (the concentration at which uptake occurs at half the maximum rate) for both extant and ancestral proteins were lower in one *Phytophthora* paralogue (paralogue 1 in fig. 1) than the other (supplementary fig. S7, Supplementary Material online), suggesting a higher affinity for malic acid, we observed too much variability in these data and in the efficiency of heterologous expression among strains for these results to be conclusive.

### Substrate Ranges Are Robust to Statistical Uncertainty

We examined the ability of nonlabeled carboxylic acids to inhibit ^14^C labeled succinic acid in four additional W303-1A *jen1*Δ *ady2*Δ strains expressing alternative ancestral proteins. The proteins contained plausible non-ML residues (posterior probabilities > 0.2) at ambiguous sites within transmembrane domains which form the putative substrate translocation pathway (supplementary fig. S5, Supplementary Material online; note that the A2 ancestral reconstruction contained no ambiguous sites in these domains and so consequently no alternative ancestral protein was synthesized for A2). This strategy, in which a single alternative protein is expressed to simultaneously account for ambiguities at multiple sites, provides an efficient approach to examine the robustness of protein function to statistical uncertainty in ancestral reconstructions (Eick et al. 2017).

The five nonlabeled dicarboxylic acids which inhibited ^14^C labeled succinic acid uptake in the strains bearing extant proteins and the primary ancestral protein forms also inhibited uptake of ^14^C labeled succinic acid in strains expressing alternative ancestral proteins A3(a) or A5(a), which correspond to the ancestor of the two *Phytophthora* paralogues and the earliest ancestral oomycete HGT transporter reconstructed, respectively (fig. 4). These results demonstrate that the putative substrate ranges of these ancestral oomycete HGT transporter proteins are robust to statistical uncertainty in the ancestral sequence reconstructions, at least within transmembrane domains that form the putative substrate translocation pathway of the transporter pore. This is consistent with previous studies showing that ancestral protein functions can be determined accurately despite uncertainty in ancestral sequences (Ortlund et al. 2007; Bar-Rogovsky et al. 2015; Randall et al. 2016; Eick et al. 2017), though occasionally phenotypic variation associated with plausible differences in ancestral sequences has been observed (e.g., Bar-Rogovsky et al. 2015; Randall et al. 2016).

Potential inhibitory effects of nonlabeled carboxylic acids could not be examined in strains expressing alternative ancestral HGT transporter proteins A1(a) or A4(a), which corresponds to the ancestor of one *Phytophthora* paralogue (paralogue 1 in fig. 1) and the ancestor of the Saprolegniales and *Pythium* clade, respectively, as ^14^C labeled succinic acid uptake was too low to distinguish from background levels of ^14^C adsorption. This suggests that these proteins are nonfunctional, at least when expressed in *S. cerevisiae* W303-1A *jen1*Δ *ady2*Δ cells, and could reflect limitations of addressing uncertainty in the ancestral sequence reconstructions using single sequences which simultaneously incorporate plausible non-ML states at all ambiguous sites within the putative substrate translocation pathway of the transporter pore. Indeed, this approach represents the “worst plausible case” scenario for each node (Eick et al. 2017), as the alternative ancestral proteins occupy positions in sequence space that are farther from the true ancestors than all other plausible ancestral sequences, when only transmembrane domains that form the putative substrate translocation pathway are taken into account. Despite this, due to the apparent lack of change in substrate specificity profiles from the most ancestral oomycete HGT transporter protein to all but one of the extant proteins that were characterized in this study, we are confident in the phenotypes observed for the primary ML protein sequences that were inferred for these ancestral nodes.

Functional characterization of the sequence space surrounding inferred ML ancestral sequences has provided important insights into the processes and constraints underlying protein evolution (Harms and Thornton, 2014; Starr and Thornton, 2016; Starr et al. 2017). For instance, a functional comparison of proteins with different combinations of amino acid substitutions revealed intraprotein epistatic interactions, whereby the effects of substitutions at sites which strongly influence protein function were dependent upon the presence of specific residues at other sites, even though these had no apparent functional effects or only weak effects when considered in isolation (Ortlund et al. 2007). Such interactions are likely to reflect structural and/or stability constraints imposed by the stochastic accumulation of historic mutations (Ortlund et al. 2007; Harms and Thornton, 2014; Starr and Thornton, 2016; Starr et al. 2017). Further functional characterization of the sequence space surrounding the primary ML ancestral proteins that were reconstructed in this study, as well as more ancient proteins that existed before the transporters were horizontally acquired, could reveal the extent to which epistatic interactions have shaped the evolutionary trajectories of the oomycete HGT transporter proteins and perhaps provide insights into the protein properties that permitted expansions in substrate ranges, such as observed for *P. aphanidermatum*.

### Possible Roles of HGT Carboxylic Acid Transporters in the Oomycetes

The confirmed oomycete HGT transporter substrates (succinic acid, malic acid and citric acid) and putative substrates which inhibited ^14^C labeled succinic acid uptake (α-ketoglutaric acid, fumaric acid, succinyl coA, and cis-aconitic acid) are all intermediates of the TCA cycle. With the exception of α-ketoglutaric acid and succinyl coA, they are also intermediates of the glyoxylate cycle, which facilitates the use of alternative carbon sources when sugars are unavailable. Oomycetes can utilize a range of mono-, di-, and tricarboxylic acids as the sole carbon source for growth, including TCA cycle and glyoxylate cycle intermediates (Chun et al. 2003; Khalil and Alsanius 2009; Van Buyten and Höfte 2013; Wang et al. 2015). Assimilation of these carboxylic acids is presumably dependent on the ability to transport them across the plasma membrane, indicating that the oomycete HGT transporter proteins could be involved in nutrient acquisition, and consistent with the suggestion that HGT contributed to the oomycete transition to an osmotrophic lifestyle from a phagotrophic and/or photosynthetic ancestral form (Richards et al. 2006; Richards and Talbot 2013). However, we cannot rule out the possibility that the oomycete HGT transporter proteins have a function that is not associated with nutrient acquisition and osmotrophy. For instance, they could play a role in cellular homeostasis, exporting rather than importing carboxylic acids in order to maintain an optimal intracellular pH. Indeed, the *S. cerevisiae* Jen1 protein is capable of lactic acid efflux (Pacheco et al. 2012). Alternatively, as a variety of carboxylic acids are present in plant root exudates (Ryan et al. 2001; Bais et al. 2006) and are presumably leaked from a range of plant and animal tissues, the HGT transporters of pathogenic oomycetes could participate in chemotaxis, facilitating the ability of motile zoospores to locate suitable hosts. Upregulation of one *Phytophthora* paralogue (paralogue 1 in fig. 1) in zoospores and/or during the cyst stage (Torto-Alalibo et al. 2007; Roy et al. 2013) is consistent with the latter possibility.

Regardless of how the function of the HGT transporters affects the ecology of the recipient microbes, the results presented here demonstrate that the substrate specificity profile of the ancestral protein has largely been retained during the evolutionary diversification of the oomycetes, even though gene duplications, paralogue losses and a possible additional case of HGT are evident. Comparative functional analysis informed by ancestral sequence reconstruction demonstrates that postacquisition divergence of the HGT transporter family has resulted in at least one major functional expansion, as observed for the *P. aphanidermatum* protein. This demonstrates the importance of functional experimentation that samples a wide diversity of the HGT acquired gene family and which investigates likely ancestral gene forms and their functions. The results therefore provide an important example of how horizontally acquired genes can add a functional phenotype, potentially playing a key role in adaptive radiations in recipient lineages, and demonstrate that transporter genes that are acquired by HGT can be subject to neofunctionalization.

## Materials and Methods

### Phylogenetic Analysis and Ancestral Sequence Reconstruction

Oomycete HGT transporter sequences and orthologues from other lineages were selected based on similarity searches (BLASTp) of protein sequences in publicly available databases. Recovered hits were filtered to remove distant paralogues and sequences representing multiple orthologues from the same nonoomycete genera. Protein sequences were aligned using MUSCLE (Edgar 2004) and the alignment was masked using Seaview version 4 (Gouy et al. 2010) (unmasked [*.mase] and masked [*.phy] alignment files are presented as supplementary data sets S1 and S2, Supplementary Material online, respectively). The best-fit model of protein evolution was determined as LG + I + Γ using ProtTest version 3.4 (Darriba et al. 2011), and a ML phylogenetic tree was generated using RaxML version 8.0.5 (Stamatakis 2014). Statistical support was evaluated with 1,000 bootstrap replicates. Phylogenetic relationships were also reconstructed using Bayesian Markov Chain Monte Carlo (MCMC) methods implemented in MrBayes version 3.2.6 (Ronquist and Huelsenbeck 2003) and PhyloBayes version 4.1 (Lartillot et al. 2009). The MrBayes analysis included four chains run for 1,000,000 MCMC generations, with trees and parameters being sampled every 100 generations. Convergence was assessed by checking the average standard deviation of split frequencies (<0.01) and the potential scale reduction factor (PSRF, close to 1.0 for all parameters). The PhyloBayes analysis was run using the CAT model (Lartillot and Philippe 2004) to improve the model accounting for evolutionary rate heterogeneity and included two chains that were stopped automatically upon convergence, when maximum discrepancies were ≤0.3 and effective sizes were over 50. For the MrBayes and PhyloBayes analyses, posterior distributions of trees were summarized after removal of 25% burnin. All phylogenetic analyses were repeated following removal of a *P. vexans* sequence from the original alignment.

Ancestral protein sequences and their posterior probability distributions were inferred from a reduced sequence alignment (supplementary data set S3, Supplementary Material online) and the corresponding ML tree (supplementary fig. S2, Supplementary Material online) using an empirical Bayes approach (Yang et al. 1995) implemented in PAML version 3.13 (Yang, 1997), assuming the ML phylogeny and the best-fit model of protein evolution. Overall confidence in the ancestral sequences reconstructions was evaluated for each node of interest by calculating mean posterior probabilities and alternative ancestral states were considered as plausible if posterior probabilities were >0.2.

### Heterologous Expression of Oomycete HGT Transporter Proteins in *S. cerevisiae*

DNA sequences coding for a selection of eight extant and five ancestral oomycete HGT transporter proteins were codon optimized for expression in *S. cerevisiae*, synthesized *de novo* (Genscript, Piscataway, NJ, USA), and inserted into a p423-GPD expression vector. Each transporter sequence contained a 6× His tag immediately before the stop codon. The vectors were used to transform a *S. cerevisiae* strain lacking native carboxylic acid transporters (W303-1A *jen1*Δ *ady2*Δ; Soares-Silva et al. 2007; kindly provided by Professor M. Casal), based on selection for complementation of a histidine auxotrophy. A negative control strain was obtained by transforming W303-1A *jen1*Δ *ady2*Δ with an empty p423-GPD vector. To generate a construct containing a confirmed dicarboxylic acid transporter as a positive control, the JEN2 open reading frame was amplified from *Candida albicans* (SC5314) genomic DNA using Phusion^®^ high-fidelity DNA polymerase (New England Biolabs, Ipswich, MA, USA) and cloned into the p423-GPD expression vector using BamHI and SalI restriction sites. This vector was used to transform the W303-1A *jen1*Δ *ady2*Δ strain, based on selection for complementation of a histidine auxotrophy.

For fluorescence microscopy, extant and ancestral oomycete open reading frames were amplified from the appropriate p423-GPD vectors with Phusion^®^ high-fidelity DNA polymerase. Amplicons were cloned into the pDONR221 vector using Gateway^®^ recombination (Life Technologies, Carlsbad, CA, USA) and mobilized into the pAG426-GPD-EGFP vector (providing an N-terminal EGFP fusion). The *Pythium aphanidermatum* open reading frame was synthesized *de novo* (Synbio-technologies Monmouth Junction, NJ, USA) and assembled into a linear pAG426-GPD-EGFP vector using SpeI and HindIII restriction sites. The pAG426-GPD-EGFP vectors were used to transform the W303-1A *jen1*Δ *ady2*Δ strain based on selection for complementation of a uracil auxotrophy.

To account for ambiguities in the ancestral sequence reconstructions, constructs were generated in which all sites with plausible alternative ancestral states (posterior probabilities >0.2) within transmembrane domains that form the putative substrate translocation pathway were simultaneously altered within a single peptide to contain the state with the second highest posterior probability. Transmembrane domains were identified based on TMpred (Hofmann and Stoffel 1993) and HMMTop (Tusnády and Simon 2001) predictions and alignment with the *S. cerevisiae* Jen1 transporter sequence (TMHMM [Krogh et al. 2001] predictions failed to detect TM XI helices with high probabilities, as previously described for Saccharomycotina Jen2 proteins and Pezizomycotina orthologues [Lodi et al. 2007]). DNA sequences coding for ancestral oomycete HGT transporter proteins were modified by site-directed mutagenesis and fragments were assembled within a p423-GPD expression vector using Gibson assembly master mix (New England Biolabs, Ipswich, MA, USA). The resulting vectors were used to transform the W303-1A *jen1*Δ *ady2*Δ strain, based on selection for complementation of a histidine auxotrophy.

Competent cells were prepared for transformations as previously described (Thompson et al. 1998), mixed with approximately 500 ng of vector DNA, and pulsed at 1.5 kV in an Eppendorf electroporator. Cells were suspended in YPD [2% bacteriological peptone (Oxoid, Milan, Italy), 1% yeast extract (Oxoid); 2% glucose] and grown at 30°C with 180 rpm shaking for 16–20 h before plating on Sc medium minus histidine (Formedium, Norfolk, UK) with 2% glucose (p423-GPD vectors), or SC medium minus uracil (Formedium, Norfolk, UK) with 2% glucose (pAG426-GPD-EGFP vectors). All strains generated in this study are listed in supplementary table S1, Supplementary Material online and primers are listed in supplementary table S2, Supplementary Material online.

### Phenotype Assays

#### Growth Assays

W303-1A *jen1*Δ *ady2*Δ strains transformed with p423-GPD vectors containing oomycete HGT transporter sequences were incubated overnight at 30°C with Sc medium minus histidine and 2% glucose. Cells were harvested by centrifugation at 2,300 rpm for 3 min, washed twice with deionized water, resuspended in synthetic complete (SC) medium minus histidine media containing 1% glucose, and adjusted to an optical density of OD_600_ = 0.2. Aliquots of 100 µl were transferred to a sterile 96 well plate, with 3–4 replicates per strain. OD_600_ measurements were recorded every 10 min for 48 h at 30°C, and growth rates were estimated from the OD_600_ measurements using a logistic population growth equation implemented using a nonlinear least squares regression in R 3.1.2 (R Core Team 2014).

#### Radiolabeled Uptake Assays

W303-1A *jen1*Δ *ady2*Δ strains transformed with p423-GPD vectors containing oomycete HGT transporter sequences were incubated overnight at 30°C with SC medium minus histidine and 2% glucose. Overnight cultures were diluted using the same media and allowed to grow at 30°C until early log phase. Cells were harvested by centrifugation at 2,300 rpm for 3 min, washed twice with deionized water, then incubated at 30°C for 2 h in SC medium minus histidine with 1% succinic, malic or citric acid (depending on the labeled acid to which they would later be exposed). Cells were harvested by centrifugation at 2,300 rpm for 3 min, washed twice with deionized water, and resuspended in 0.1 M potassium phosphate buffer (pH 5.0) at a concentration of approximately 15–25 mg dry weight ml^−1^. To start the uptake reactions, 90 µl aliquots of the cell suspensions were mixed with 10 µl of radiolabeled carboxylic acid at various concentrations and incubated at 30°C for 1 min (Soares-Silva et al. 2007). Different molar concentrations were obtained by combining ^14^C labeled substrates with nonlabeled counterparts and adjusting the specific activity accordingly using a specific activity adjustment calculator (www.perkinelmer.co.uk). The following radiolabeled carboxylic acids were used: [2, 3-^14^C] succinic acid (American Radiolabeled Chemicals, St. Louis, USA), L-[U-^14^C] malic acid (PerkinElmer, Wokingham, UK) and [1, 5-^14^C] citric acid (PerkinElmer, Wokingham, UK). Uptake reactions were stopped by adding 1 ml of ice-cold 120 mM nonlabeled succinic, malic or citric acid (pH 5.0). Background adsorption of ^14^C was determined by exposing cells to 1 ml of 120 mM nonlabeled succinic, malic or citric acid (pH 5.0) prior to the addition of the ^14^C labeled acid. Cells were centrifuged at 13,000 rpm for 3 min, washed in 1 ml deionized water, resuspended in 0.5 ml deionized water then added to scintillation vials containing 2.5 ml scintillation fluid (PerkinElmer, Wokingham, UK). Radioactivity was measured in a Packard Tri-Carb 2200 CA liquid scintillation counter. Uptake assays were repeated 3–4 times for each radiolabeled substrate and strain. Kinetic parameters were estimated in R 3.1.2 using the Dose-Response Model (drm) function in the DRC library (Ritz et al. 2015).

The ability of alternative substrates to inhibit succinic acid uptake was assessed by simultaneously exposing cells prepared as described above to ^14^C labeled succinic acid (50 µM) and nonlabeled substrates (50 mM). The nonlabeled substrates included seven monocarboxylic acids (acetic, formic, D-galacturonic, D-gluconic, lactic, propionic, and pyruvic acid), ten dicarboxylic acids (α-ketoglutaric, fumaric, maleic, malic, malonic, oxalic, oxaloacetic, succinic, and tartaric acid, as well as succinyl coA, a combination of succinic acid and coenzyme A), three tricarboxylic acids (cis-aconitic, citric, and DL-isocitric acid) and glucose. Uptake reactions were stopped by adding 1 ml of ice-cold 120 mM nonlabeled succinic acid (pH 5.0) and cells were prepared for scintillation counting as described above. Radioactivity values were then compared with those from control cells that had been exposed to ^14^C labeled succinic acid (50 µM) with no additional nonlabeled substrate added and were expressed as percentage change. Whilst negative values indicate inhibition, positive values indicate enhanced uptake. Inhibition assays were repeated 3–5 times for each nonlabeled substrate and strain. Due to variation across replicates, we classified a nonlabeled substrate as a moderate inhibitor or a strong inhibitor of ^14^C succinic acid uptake if the upper quartile of the response was <−50% or <−80%, respectively.

### Spinning Disc Confocal Microscopy

*Saccharomyces cerevisiae* strains transformed with pAG426-GPD-EGFP vectors were grown in SC medium minus uracil with 2% glucose at 30 °C with 180 rpm shaking to mid-log phase, then were suspended in PBS containing 10 µl/ml Wheat Germ Agglutinin, Alexa Fluor 594 Conjugate (WGA, Life Technologies) and incubated at room temperature in the dark for 1–2 h. Spinning disc confocal microscopy was performed using an Olympus IX81 inverted microscope and CSU-X1 Spinning Disc unit (Yokogawa, Tokyo, Japan). A ×100/1.40 oil objective was used with a 488 nm solid-state laser to excite the EGFP fluorophore and a 594 nm solid-state laser to visualize the WGA stain. A Photometrics CoolSNAP HQ2 camera (Roper Scientific, Martinsried, Germany) was used for imaging with the VisiView software package (Visitron Systems, Puchheim, Germany).

### Flow Cytometry

*Saccharomyces cerevisiae* strains transformed with pAG426-GPD-EGFP vectors were grown in SC medium minus uracil with 2% glucose at 30°C with 180 rpm shaking to mid-log phase, then were suspended in PBSE (10 mM Na_2_HPO_4_, 2 mM KH_2_PO_4_, 137 mM NaCl, 2.7 mM KCl, 0.1 mM EDTA, pH 7.4) containing 1 µg/ml propidium iodide. Three samples for each strain were run on a CytoFLEX S flow cytometer (Beckman Coulter), and proportions of live cells expressing GFP-transporter fusion proteins were determined using CytExpert.

## Supplementary Material

Supplementary data are available at *Molecular Biology and Evolution* online.

## Supplementary Material

Supplementary Data
