# Supplementary material for: Ancestral Function and Diversification of a Horizontally Acquired Oomycete Carboxylic Acid Transporter
Source: Mol Biol Evol. 2018 Apr 25;35(8):1887–900. doi: 10.1093/molbev/msy082 (PMC6063262; doi:10.1093/molbev/msy082)
Supplement: Supplementary Data [file msy082_supp.zip › Supplementary Tables.pdf]

Table S1 – *S. cerevisiae* strains used in this study

| Strain | Genotype | Vector(s) | Reference |
|---|---|---|---|
| W303-1A Δ*jen1* Δ*ady2* | MATa, *can1-100, ade2-1*$^{oc}$*, his3-11-15, leu2-3,-112, trp1-1-1, ura3-1, jen1::kanMX4, ady2::hphMX4* | n/a | Soares-Silva et al. 2007 |
| W303-1A Δ*jen1* Δ*ady2* p423-GPD empty vector | As W303-1A Δ*jen1* Δ*ady2* | p423-GPD empty vector | This study |
| W303-1A Δ*jen1* Δ*ady2* *P. infestans* (1) | As W303-1A Δ*jen1* Δ*ady2* + vector | p423-GPD + codon optimised *P. infestans* (paralogue 1) transporter | This study |
| W303-1A Δ*jen1* Δ*ady2* *P. parasitica* (1) | As W303-1A Δ*jen1* Δ*ady2* + vector | p423-GPD + codon optimised *P. parasitica* (paralogue 1) transporter | This study |
| W303-1A Δ*jen1* Δ*ady2* *H. arabidopsidis* | As W303-1A Δ*jen1* Δ*ady2* + vector | p423-GPD + codon optimised *H. arabidopsidis* transporter | This study |
| W303-1A Δ*jen1* Δ*ady2* *P. infestans* (2) | As W303-1A Δ*jen1* Δ*ady2* + vector | p423-GPD + codon optimised *P. infestans* (paralogue 2) transporter | This study |
| W303-1A Δ*jen1* Δ*ady2* *P. parasitica* (2) | As W303-1A Δ*jen1* Δ*ady2* + vector | p423-GPD + codon optimised *P. parasitica* (paralogue 2) transporter | This study |
| W303-1A Δ*jen1* Δ*ady2* *P. aphanidermatum* | As W303-1A Δ*jen1* Δ*ady2* + vector | p423-GPD + codon optimised *P. aphanidermatum* transporter | This study |
| W303-1A Δ*jen1* Δ*ady2* *S. declina* | As W303-1A Δ*jen1* Δ*ady2* + vector | p423-GPD + codon optimised *S. declina* transporter | This study |
| W303-1A Δ*jen1* Δ*ady2* *T. clavata* | As W303-1A Δ*jen1* Δ*ady2* + vector | p423-GPD + codon optimised *T. clavata* transporter | This study |
| W303-1A Δ*jen1* Δ*ady2* A1 | As W303-1A Δ*jen1* Δ*ady2* + vector | p423-GPD + codon optimised A1 transporter | This study |
| W303-1A Δ*jen1* Δ*ady2* A2 | As W303-1A Δ*jen1* Δ*ady2* + vector | p423-GPD + codon optimised A2 transporter | This study |
| W303-1A Δ*jen1* Δ*ady2* A3 | As W303-1A Δ*jen1* Δ*ady2* + vector | p423-GPD + codon optimised A3 transporter | This study |
| W303-1A Δ*jen1* Δ*ady2* A4 | As W303-1A Δ*jen1* Δ*ady2* + vector | p423-GPD + codon optimised A4 transporter | This study |
| W303-1A Δ*jen1* Δ*ady2* A5 | As W303-1A Δ*jen1* Δ*ady2* + vector | p423-GPD + codon optimised A5 transporter | This study |
| W303-1A Δ*jen1* Δ*ady2* A1.2 | As W303-1A Δ*jen1* Δ*ady2* + vector | p423-GPD + codon optimised A1.2 transporter | This study |
| W303-1A Δ*jen1* Δ*ady2* A3.2 | As W303-1A Δ*jen1* Δ*ady2* + vector | p423-GPD + codon optimised A3.2 transporter | This study |
| W303-1A Δ*jen1* Δ*ady2* A4.2 | As W303-1A Δ*jen1* Δ*ady2* + vector | p423-GPD + codon optimised A4.2 transporter | This study |
| W303-1A Δ*jen1* Δ*ady2* A5.2 | As W303-1A Δ*jen1* Δ*ady2* + vector | p423-GPD + codon optimised A5.2 transporter | This study |
| W303-1A Δ*jen1* Δ*ady2* *P. infestans* (1) GFP | As W303-1A Δ*jen1* Δ*ady2* + vector | pAG426-GPD-EGFP + codon optimised *P. infestans* (paralogue 1) transporter | This study |
| W303-1A Δ*jen1* Δ*ady2* *P. parasitica* (1) GFP | As W303-1A Δ*jen1* Δ*ady2* + vector | pAG426-GPD-EGFP + codon optimised *P. parasitica* (paralogue 1) transporter | This study |
| W303-1A Δ*jen1* Δ*ady2* *H. arabidopsidis* GFP | As W303-1A Δ*jen1* Δ*ady2* + vector | pAG426-GPD-EGFP + codon optimised *H. arabidopsidis* transporter | This study |
| W303-1A Δ*jen1* Δ*ady2* *P. infestans* (2) GFP | As W303-1A Δ*jen1* Δ*ady2* + vector | pAG426-GPD-EGFP + codon optimised *P. infestans* (paralogue 2) transporter | This study |
| W303-1A Δ*jen1* Δ*ady2* *P. parasitica* (2) GFP | As W303-1A Δ*jen1* Δ*ady2* + vector | pAG426-GPD-EGFP + codon optimised *P. parasitica* (paralogue 2) transporter | This study |
| W303-1A Δ*jen1* Δ*ady2* *P. aphanidermatum* GFP | As W303-1A Δ*jen1* Δ*ady2* + vector | pAG426-GPD-EGFP + codon optimised *P. aphanidermatum* transporter | This study |
| W303-1A Δ*jen1* Δ*ady2* *S. declina* GFP | As W303-1A Δ*jen1* Δ*ady2* + vector | pAG426-GPD-EGFP + codon optimised *S. declina* transporter | This study |
| W303-1A Δ*jen1* Δ*ady2* *T. clavata* GFP | As W303-1A Δ*jen1* Δ*ady2* + vector | pAG426-GPD-EGFP + codon optimised *T. clavata* transporter | This study |
| W303-1A Δ*jen1* Δ*ady2* A1 GFP | As W303-1A Δ*jen1* Δ*ady2* + vector | pAG426-GPD-EGFP + codon optimised A1 transporter | This study |
| W303-1A Δ*jen1* Δ*ady2* A2 GFP | As W303-1A Δ*jen1* Δ*ady2* + vector | pAG426-GPD-EGFP + codon optimised A2 transporter | This study |
| W303-1A Δ*jen1* Δ*ady2* A3 GFP | As W303-1A Δ*jen1* Δ*ady2* + vector | pAG426-GPD-EGFP + codon optimised A3 transporter | This study |
| W303-1A Δ*jen1* Δ*ady2* A4 GFP | As W303-1A Δ*jen1* Δ*ady2* + vector | pAG426-GPD-EGFP + codon optimised A4 transporter | This study |
| W303-1A Δ*jen1* Δ*ady2* A5 GFP | As W303-1A Δ*jen1* Δ*ady2* + vector | pAG426-GPD-EGFP + codon optimised A5 transporter | This study |

Table S2 – Primers used in this study

| Method | Transporter | Forward primer | Reverse primer |
|---|---|---|---|
| **Gateway cloning** | *P. infestans* (paralogue 1) | GGGGACAAGTTTGTACAAAAAAGCAGGCTTGATGCCTTCCGTTACCGATT | GGGGACCACTTTGTACAAGAAAGCTGGGTCAGCCTTAAGGGACTTTGCCT |
| | *P. parasitica* (paralogue 1) | GGGGACAAGTTTGTACAAAAAAGCAGGCTTGATGCCATCCGTTACTGATT | GGGGACCACTTTGTACAAGAAAGCTGGGTCGGCTTTCATACTTTTTGTGC |
| | *H. arabidopsidis* | GGGGACAAGTTTGTACAAAAAAGCAGGCTTGATGCCATCATATCAGGTCG | GGGGACCACTTTGTACAAGAAAGCTGGGTCAGCCTTCATGGACTTTGTTT |
| | *P. infestans* (paralogue 2) | GGGGACAAGTTTGTACAAAAAAGCAGGCTTGATGGCACCAGTTCCAGAAA | GGGGACCACTTTGTACAAGAAAGCTGGGTCACCTTGTTTAGTCGCTTTAT |
| | *P. parasitica* (paralogue 2) | GGGGACAAGTTTGTACAAAAAAGCAGGCTTGATGGCCCCAGTCCCTCAAT | GGGGACCACTTTGTACAAGAAAGCTGGGTCACCTTGTTTTGTTGTCTTAT |
| | *S. declina* | GGGGACAAGTTTGTACAAAAAAGCAGGCTTGATGGCCTCAACCCCCAAAA | GGGGACCACTTTGTACAAGAAAGCTGGGTCCATTTTCGGCTTTTCCAAAT |
| | *T. clavata* | GGGGACAAGTTTGTACAAAAAAGCAGGCTTGATGATTTCCCCAAAAGCTG | GGGGACCACTTTGTACAAGAAAGCTGGGTCTTGAGTAGTGAAGGCAACGG |
| | A1 | GGGGACAAGTTTGTACAAAAAAGCAGGCTTGATGCCCTCCAAGTCTGACT | GGGGACCACTTTGTACAAGAAAGCTGGGTCAGCTTTTGCTGTCCCACCCT |
| | A2 | GGGGACAAGTTTGTACAAAAAAGCAGGCTTGATGGCCCCTGTTCCACAGT | GGGGACCACTTTGTACAAGAAAGCTGGGTCGCCACCTTTAACTGTTTTAT |
| | A3 | GGGGACAAGTTTGTACAAAAAAGCAGGCTTGATGGCCCCGCAATCACCAA | GGGGACCACTTTGTACAAGAAAGCTGGGTCTACTTTAGCACCACCTTTTA |
| | A4 | GGGGACAAGTTTGTACAAAAAAGCAGGCTTGATGGCACCTCAAAGTCCAA | GGGGACCACTTTGTACAAGAAAGCTGGGTCAACTTTGGCTCCACCTTTTA |
| | A5 | GGGGACAAGTTTGTACAAAAAAGCAGGCTTGATGGCACCTCAAAGTCCAA | GGGGACCACTTTGTACAAGAAAGCTGGGTCTACTCTAGCTCCACCTTTGA |
| **Gibson assembly** | A1.2 (fragment 1) | GATTCTAGAACTAGTGGATCCATGCCCTCCAAG | GACCAGGTAGCACAATAACATCCAATGTCTCTTAAG |
| | A1.2 (fragment 2) | CATTGGATGTTATTGTGCTACCTGGTCTTGT | ACATGACTCGAGGTCGACTTAATGATGATG |
| | A3.2 (fragment 1) | GATTCTAGAACTAGTGGATCCATGGC | GCTGCAATACCGAATATGATTGATC |
| | A3.2 (fragment 2) | GGATCAATCATATTCGGTATTGCAGCAG | GACCAAGTAGCATAACAACATCCAATGACG |
| | A3.2 (fragment 3) | TGGATGTTGTTATGCTACTTGGTCCTATTAATGG | ACATGACTCGAGGTCGACTTAATGATGATG |
| | A4.2 (fragment 1) | GATTCTAGAACTAGTGGATCCATGGCACCTC | AGCCGATGGATCTTAGCATTAATGTTATAGTGATTC |
| | A4.2 (fragment 2) | TGCTAAGATCCATCGGCTCAATCATCTTTG | CCAGCCATAAAAAGTACTAGATAACATAGCAACAACCAG |
| | A4.2 (fragment 3) | CTAGTACTTTTTATGGCTGGATTTAATTTCATGTCTCAC | ACATGACTCGAGGTCGACTTAATGATGATG |
| | A5.2 (fragment 1) | GATTCTAGAACTAGTGGATCCATGGCACCTC | GCAACAACCAATGACGTTTTAAAGCCACT |
| | A5.2 (fragment 2) | GTGGCTTTAAAACGTCATTGGTTGTTGCTAGTTTATATGG | TTGGTAGGAGGTCCCAACTACGAAGGTACGGTAAGA |
| | A5.2 (fragment 3) | TTGGGACCTCCTACCAACTGGGGAATCTGGTTTCT | ACATGACTCGAGGTCGACTTAATGATGATGATGATGATG |
| **Other** | *C. albicans* JEN2 | CCGGGATCCGAAAATATGACTGCTGCTGATACTC | CGCGTCGACTTAATGATGATGATGATGATGCTCTTTATGTTCAACTTCT |
