# Supplementary material for: Ancestral Function and Diversification of a Horizontally Acquired Oomycete Carboxylic Acid Transporter
Source: Mol Biol Evol. 2018 Apr 25;35(8):1887–900. doi: 10.1093/molbev/msy082 (PMC6063262; doi:10.1093/molbev/msy082)
Supplement: Supplementary Data [file msy082_supp.zip › Supplementary_Figures_updated.pdf]

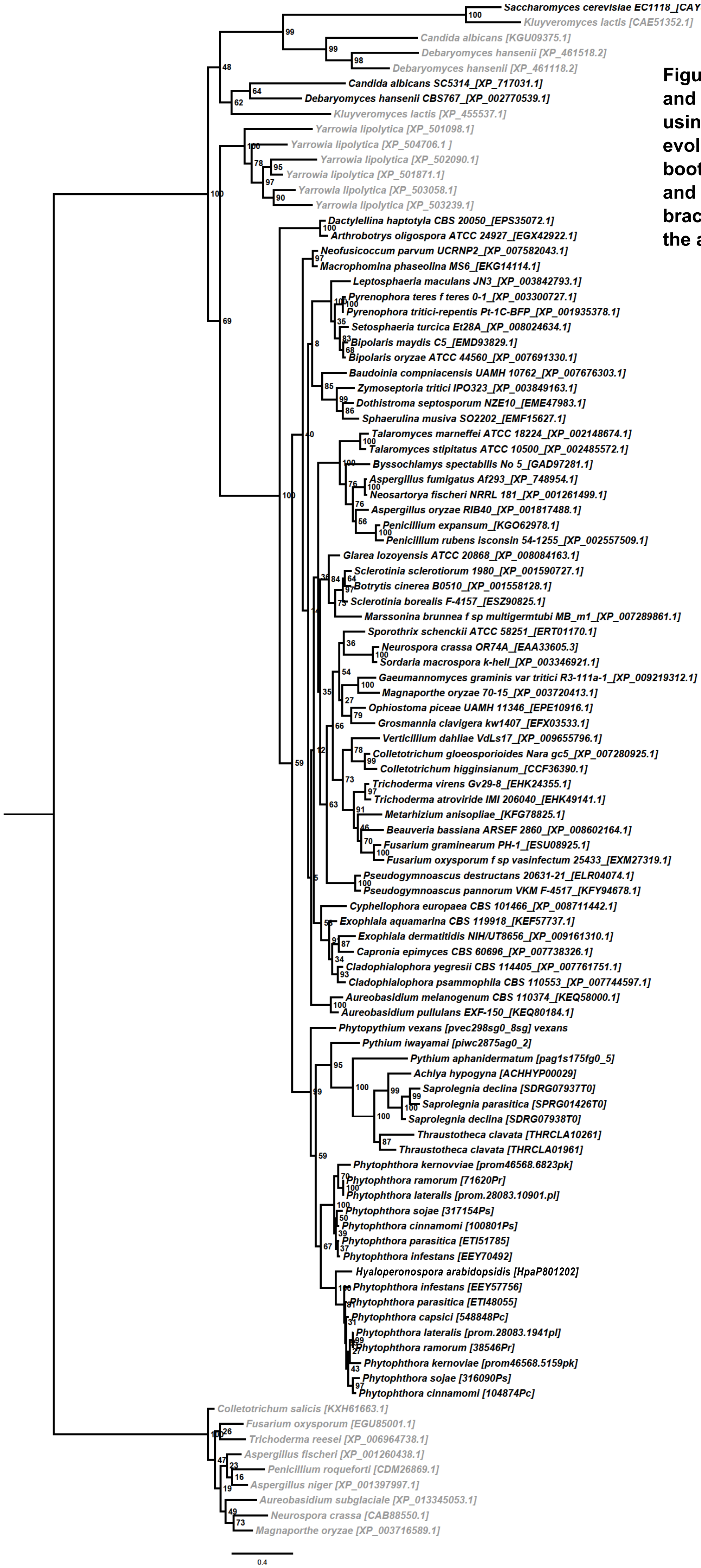

Figure S1. RaxML phylogeny of oomycete and fungal transporter proteins, generated using the LG + I + Γ model of protein evolution. Node values correspond to bootstrap support (1000 bootstrap replicates) and accession numbers are displayed in brackets. Taxa in grey were excluded from the ancestral sequence reconstruction.

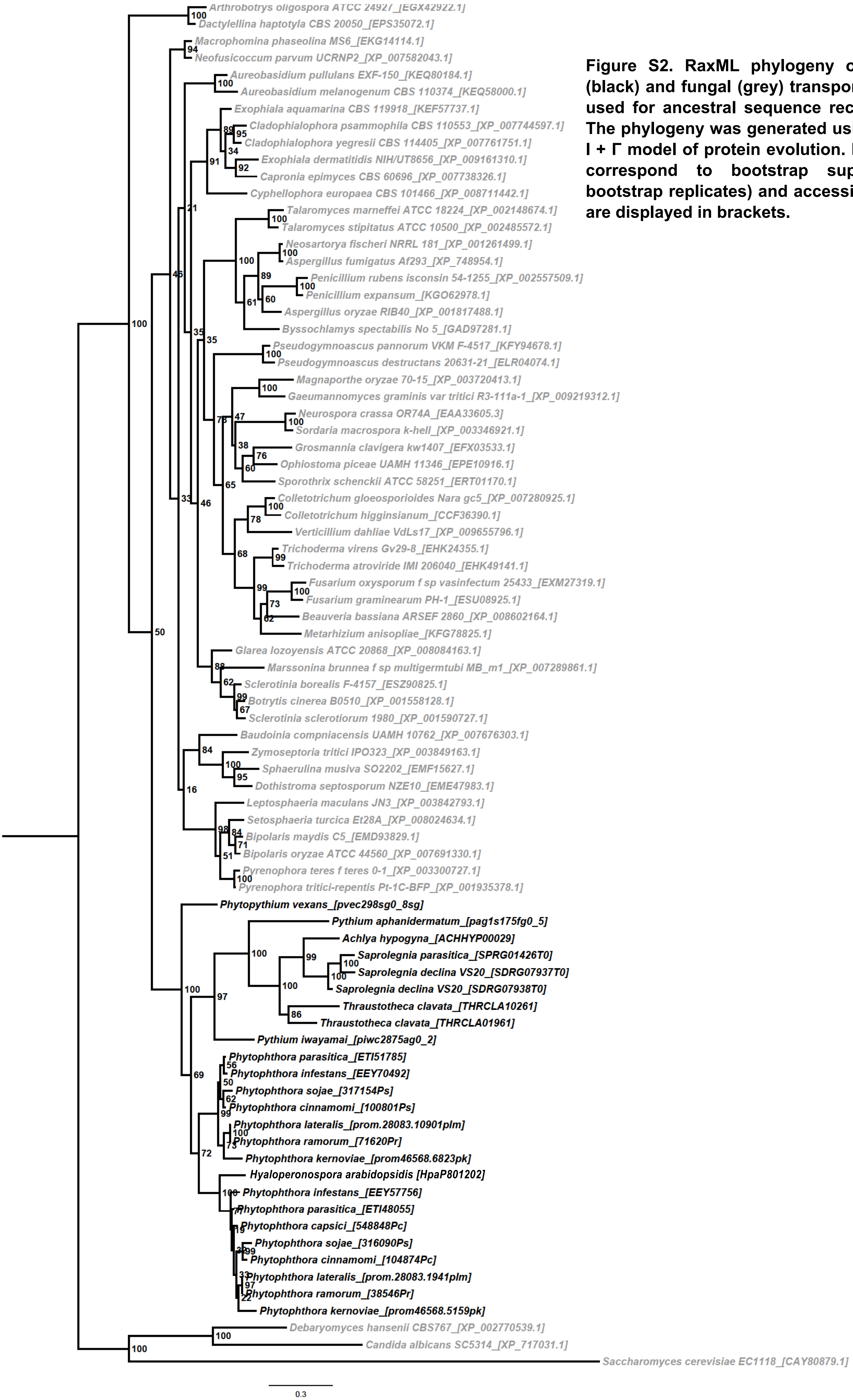

Figure S2. RaxML phylogeny of oomycete (black) and fungal (grey) transporter proteins used for ancestral sequence reconstruction. The phylogeny was generated using the LG + I + Γ model of protein evolution. Node values correspond to bootstrap support (1000 bootstrap replicates) and accession numbers are displayed in brackets.

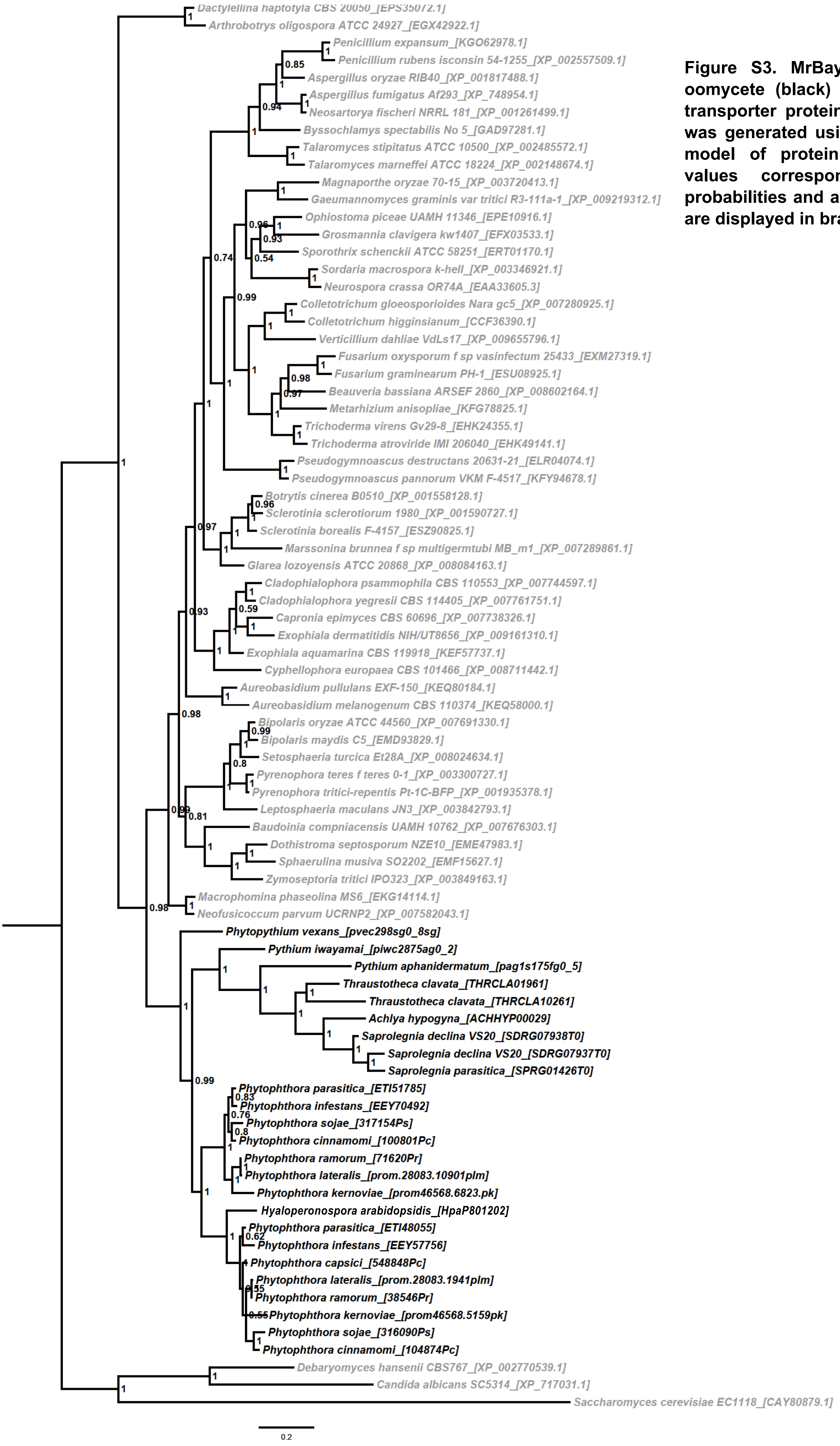

Figure S3. MrBayes phylogeny of oomycete (black) and fungal (grey) transporter proteins. The phylogeny was generated using the LG + I + Γ model of protein evolution. Node values correspond to posterior probabilities and accession numbers are displayed in brackets.

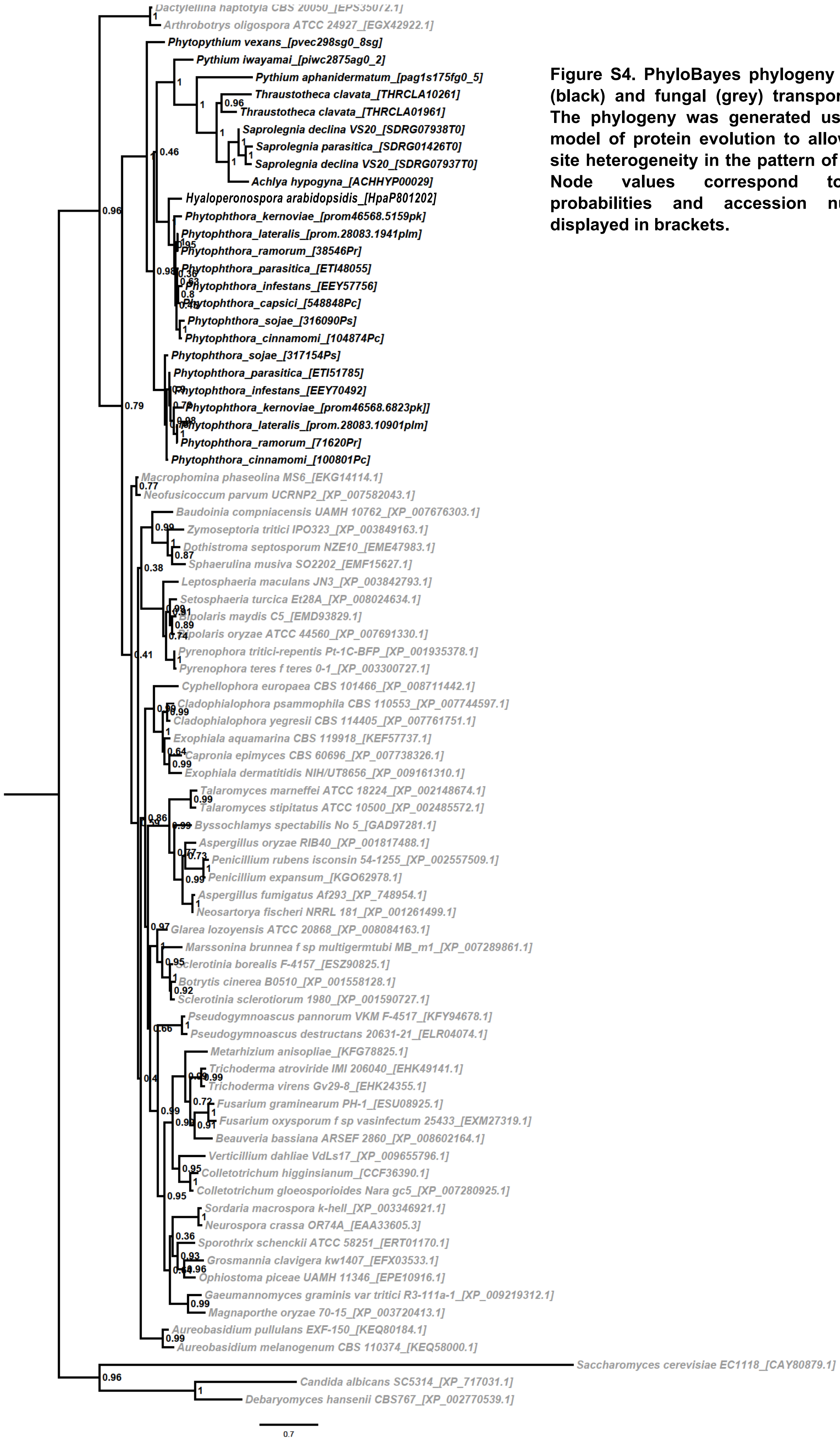

Figure S4. PhyloBayes phylogeny of oomycete (black) and fungal (grey) transporter proteins. The phylogeny was generated using the CAT model of protein evolution to allow for among site heterogeneity in the pattern of substitution. Node values correspond to posterior probabilities and accession numbers are displayed in brackets.

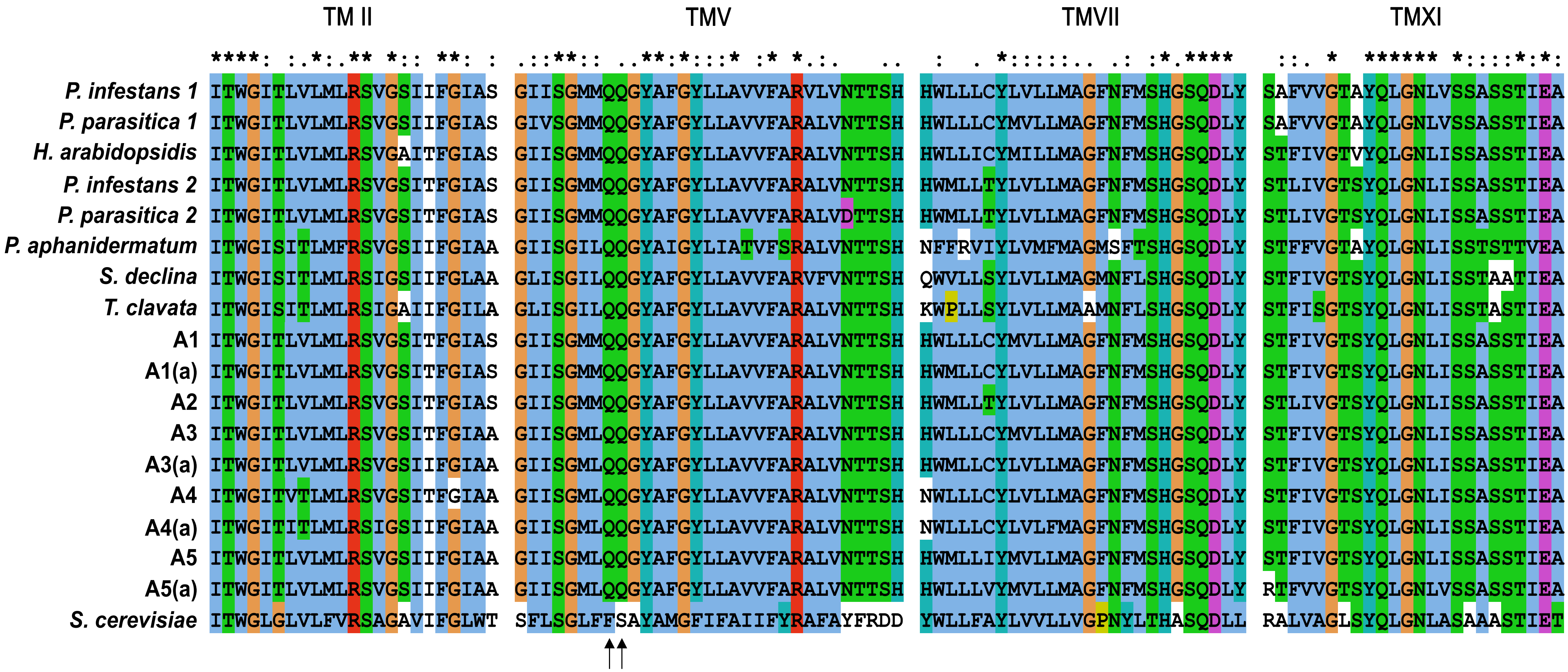

Figure S5. Alignment of transmembrane (TM) domains that form the putative substrate translocation pathway. The alignment contains TM domain sequences for all characterised extant and ancestral oomycete HGT transporter proteins, including ancestral proteins with alternative states at ambiguous sites (A1(a), A3(a), A4(a) and A5(a)). The *S. cerevisiae* JEN1 TM domain sequences are included for reference. Symbols correspond to the level of residue conservation among the displayed sequences only (asterisk - fully conserved; colon - highly conserved; period - weakly conserved), and arrows indicate residues which are critical for the distinction of monocarboxylic acids from dicarboxylic acids (Soares-Silva *et al.*, 2011).

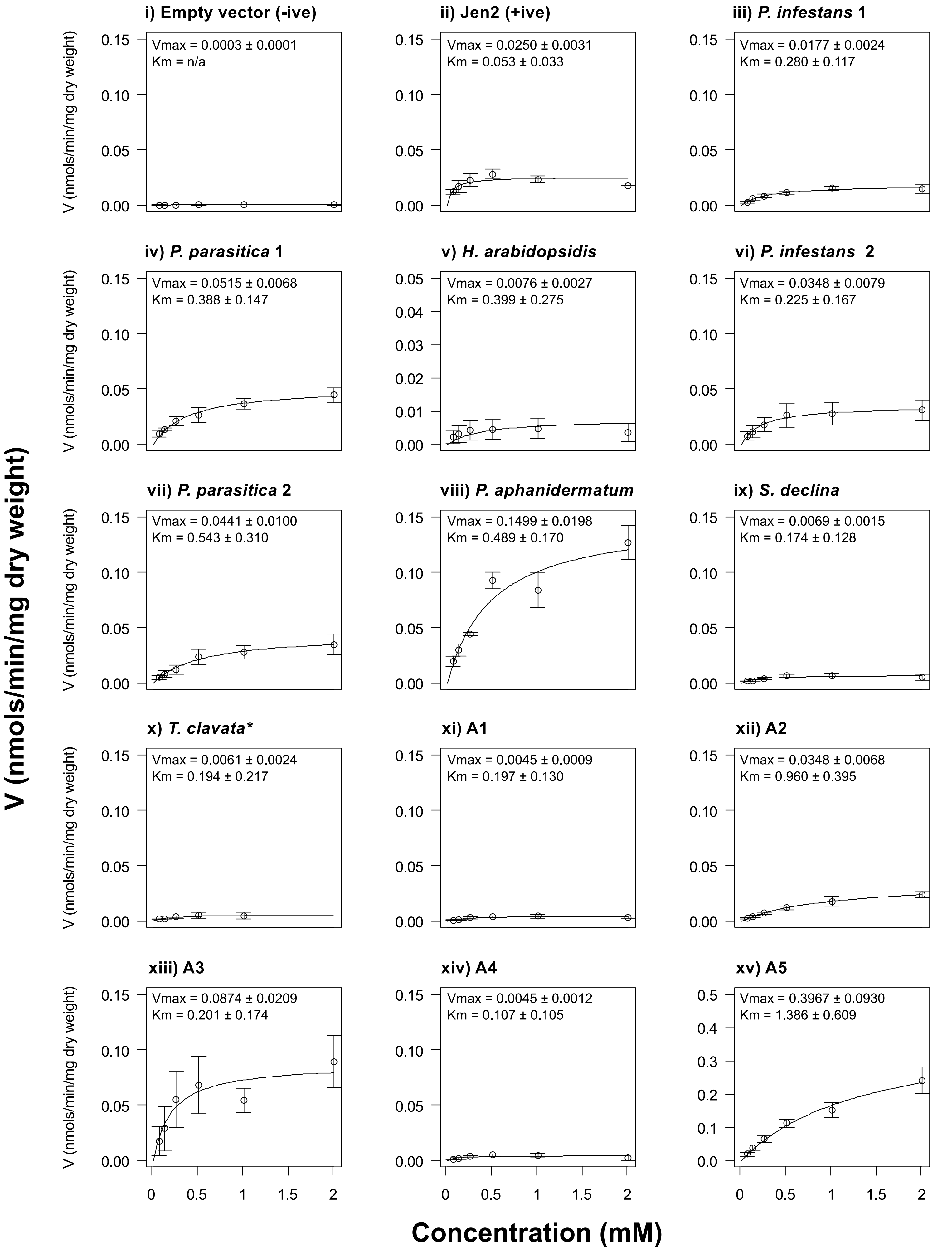

Figure S6. Initial uptake rates of different concentrations of radiolabelled ^{14}C-succinic acid (pH 5.0, 30°C) by *S. cerevisiae* W303-1A *jen1*Δ *ady2*Δ cells transformed with an empty vector (i), expressing the *C. albicans* JEN2 transporter (ii), or expressing extant (iii-x) or ancestral (x-xv) oomycete HGT transporter proteins (note differences in y-axis values). Vmax and Km values are displayed with standard errors (data are displayed from three independent experiments). (T. clavata* uptake rates are not displayed for 2 mM ^{14}C-succinic acid due to an inability to distinguish uptake from background ^{14}C adsorption in two replicates).**

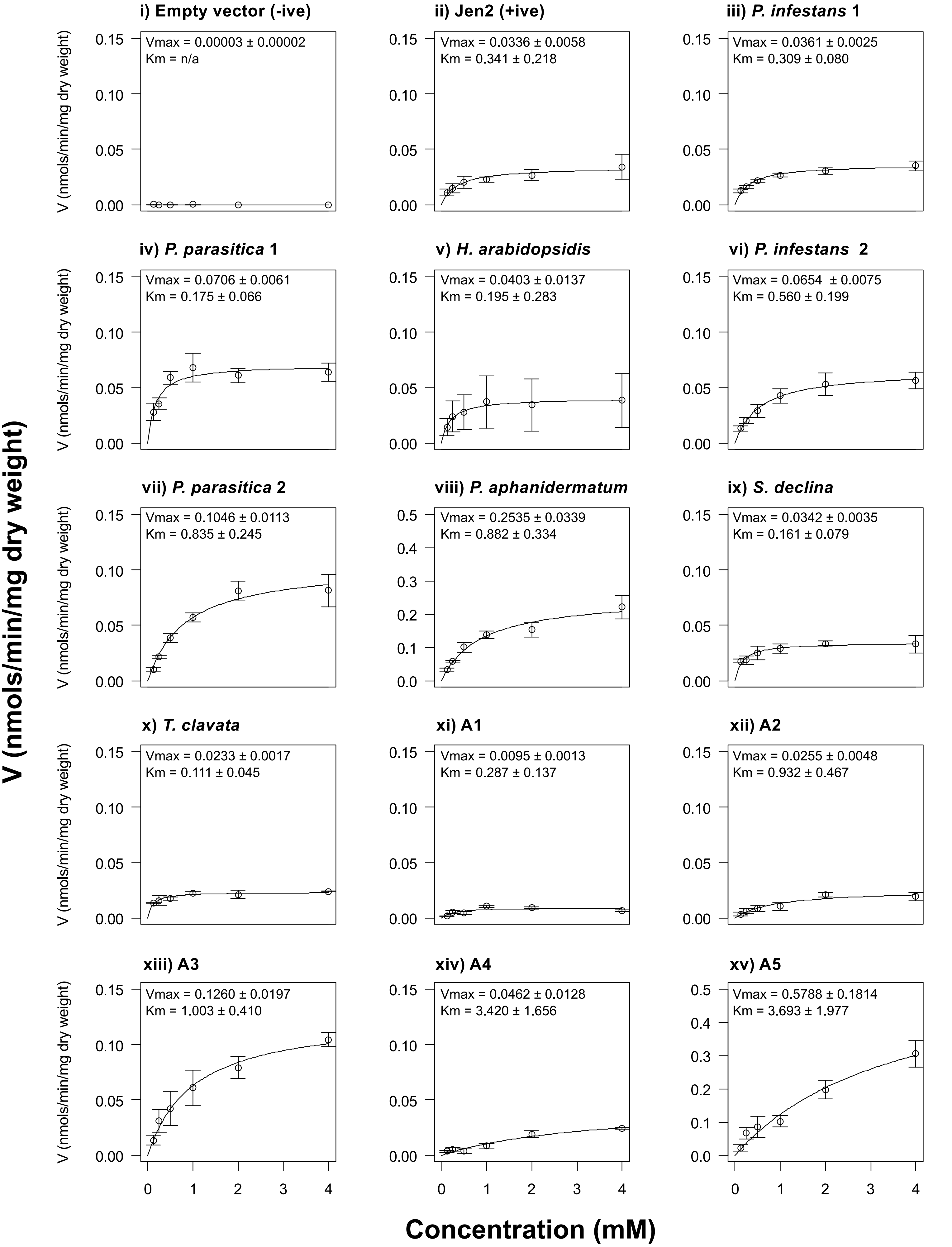

Figure S7. Initial uptake rates of different concentrations of radiolabelled ^{14}C-malic acid (pH 5.0, 30°C) by *S. cerevisiae* W303-1A jen1Δ ady2Δ cells transformed with an empty vector (i), expressing the *C. albicans* JEN2 transporter (ii), or expressing extant (iii-x) or ancestral (x-xv) oomycete HGT transporter proteins (note differences in y-axis values). Vmax and Km values are displayed with standard errors (data are displayed from at least 3 independent experiments).
